# Association between statins and infections among patients with diabetes: a cohort and prescription sequence symmetry analysis

**DOI:** 10.1002/pds.4052

**Published:** 2016-06-30

**Authors:** Koen B. Pouwels, Niken N. Widyakusuma, Jens H. J. Bos, Eelko Hak

**Affiliations:** ^1^ Unit of Pharmacoepidemiology and Pharmacoeconomics, Department of PharmacyUniversity of GroningenGroningenthe Netherlands

**Keywords:** hydroxymethylglutaryl‐CoA reductase inhibitors, cohort studies, antibiotics, Diabetes mellitus, infection, prescription sequence symmetry analysis, pharmacoepidemiology

## Abstract

**Purpose:**

A previous meta‐analysis of randomized trials did not confirm findings from observational studies that suggested that statins reduce the risk of infection. However, animal experiments indicate that statins may be more effective in reducing the risk and/or the severity of infection among patients with diabetes. Hence, we evaluated the effect of statins on antibiotic prescriptions (a proxy for infections) among patients with drug‐treated type 2 diabetes using two confounding‐reducing observational designs.

**Methods:**

We conducted a prescription sequence symmetry analysis and a cohort study using the IADB.nl pharmacy prescription database. For the prescription sequence symmetry analysis, a sequence ratio was calculated. The matched cohort study, comparing the time to first antibiotic prescription between periods that statins are initiated and non‐use periods, was analyzed using stratified Cox regression.

**Results:**

Prescription sequence symmetry analysis of 4684 patients with drug‐treated type 2 diabetes resulted in an adjusted sequence ratio of 0.86 (95% confidence interval [CI]: 0.81 to 0.91). Corresponding figures for the cohort analysis comparing 9852 statin‐initiation with 4928 non‐use periods showed similar results (adjusted hazard ratio: 0.88, 95%CI: 0.83 to 0.95).

**Conclusions:**

These findings suggest that statins are associated with a reduced risk of infections among patients with drug‐treated type 2 diabetes. © 2016 The Authors. *Pharmacoepidemiology and Drug Safety* Published by John Wiley & Sons Ltd.

## Introduction

Evidence from several observational studies suggest that statins reduce the severity and risk of various infections, including sepsis, pneumonia, hospital‐acquired infections, community‐acquired infections, influenza, bacteremia, and urinary tract infections.^1^, ^2^, ^3^, ^4^ However, a meta‐analysis of data from large randomized statin trials did not show a reduced risk of infections among the treated group.^5^ Unmeasured confounding may explain the discrepancy between randomized and non‐randomized studies.^6^, ^7^

On the other hand, all of the randomized trials included in the meta‐analysis were primarily designed to evaluate the effect of statins on cardiovascular outcomes; hence, the risk of infection could be measured only post hoc. The huge variation in the incidence of infections among the different trials^5^ suggests that the validity of the reported infectious outcomes among the included trials may have differed.

Even when statins do not have a beneficial influence on the risk of infections in the general population at low risk for infections, it is still possible that statins lower this risk in certain high‐risk subgroups, such as patients with diabetes.^7^, ^8^ Diabetes is associated with an increased formation of biofilms by different bacteria and fungi.^9^, ^10^, ^11^ Formation of such biofilms hinders the clearance of these pathogens by the immune system and thus increases the likelihood that antimicrobial agents are administered to treat an infection. A recent case–control study found that diabetes strongly increases the risk of biofilm‐forming Candida bloodstream infection but was not a risk factor for non‐biofilm‐forming Candida bloodstream infections.^11^ Hence, drugs reducing the likelihood of biofilm formation may be more effective among patients with diabetes than patients without diabetes. Simvastatin possesses pronounced antimicrobial activity against *Staphylococcus aureus* biofilms,^12^ suggesting that statins may reduce the risk of infection or reduce the need to use antibiotic treatment among patients with diabetes.

Statins are also known to reduce the amount of membrane‐associated or activated Rac1,^13^, ^14^, ^15^ a Rho GTPase involved in bacterial invasion.^16^, ^17^, ^18^, ^19^ This activity against Rac1 may explain the observation in pre‐clinical studies that statins reduce bacterial invasion.^20^, ^21^ Several studies indicate that diabetes is associated with increased Rac1 activation,^22^, ^23^ suggesting that statins should be particularly effective among patients with diabetes. Among patients without diabetes, the potentially smaller benefits may be outweighed by the increased risk of incident diabetes among patients using statins.^24^

Some observational studies with conventional cohort and case–control designs suggest that statins may reduce the risk of infection among patients with drug‐treated type 2 diabetes, but unmeasured confounding could not be ruled out.^25^, ^26^, ^27^, ^28^, ^29^ Hence, we aimed to evaluate whether statins reduce the risk of receiving first antibiotic prescriptions (as a proxy for infections) among patients with drug‐treated type 2 diabetes using two observational studies that are designed to address confounding in different ways: a prescription sequence symmetry analysis (PSSA) and a matched cohort study. In secondary analysis, we evaluated whether the effect among patients without drug‐treated diabetes would be less pronounced.

## Materials and Methods

This study was performed with the University of Groningen IADB.nl pharmacy prescription database, which contains prescription data collected from community pharmacies and covers an estimated population of 600 000 persons in the Netherlands (www.iadb.nl). Age, gender, and prescription rates among the database population have been found to be representative for the Netherlands as a whole.^30^ Each participant has a unique anonymous identifier; date of birth and gender are known. The medication records of each patient are virtually complete, except for over the counter drugs and medication dispensed during hospitalization.^30^ Data between January 1999 and December 2012 were used for the analyses.

### Prescription sequence symmetry analysis

We first applied a PSSA, a case‐only design that has powerful properties to control for genetic and other time invariant confounding.^31^, ^32^, ^33^, ^34^, ^35^ Because only patients that have an indication to initiate the treatment of interest are included and all of them experience the outcome of interest close to the date of treatment initiation, confounding between study subjects is minimized. To be able to apply this design, individuals initiating both statin treatment (ATC codes C10AA and C10B) and antibiotic treatment (ATC code J01) between January 1999 and December 2012 were selected from the IADB.nl database. Patients who initiated antibiotics and statins on the same date were excluded. Patients were only included in the study when they were present in the database for a least 13 months before initiation of statin or antibiotic treatment (whichever came first). Furthermore, we required patients to be 45–80 years old at the date of statin initiation, an age range that agrees with the previously mentioned meta‐analysis.^5^ Moreover, the majority of statin initiators will fall in this age category. Patients with drug‐treated type 2 diabetes were selected by the use of two prescriptions of non‐insulin blood‐glucose lowering drugs (ATC code A10B) within 12 months before the date of statin initiation. We excluded patients on insulin monotherapy, because the majority of those patients have type 1 diabetes. This implies that approximately 6% of type 2 diabetes patients that are only treated with insulin are excluded from the current study.^36^ As we restricted our population to 45–80 years old, misclassification because of metformin use for polycystic ovary syndrome is not likely an important source of bias. Subsequently, the PSSA was performed to evaluate whether antibiotics were more initiated in the 13 months prior statin initiation than the 13 months after statin initiation. By dividing the number of patients that first initiate statins and then antibiotics by the number of patients that first initiate antibiotics before initiating statin treatment the sequence ratio (SR) is obtained. We adjusted for trends in prescribing by adjusting the SR by dividing by the null ratio.^31^, ^32^, ^33^, ^34^ We estimated 95% confidence intervals (CIs) using a normal approximation to the binomial distribution.

### Matched cohort design

Second, we applied a matched cohort design. We used the same inclusion and exclusion criteria as in the PSSA. However, instead of selecting only patients initiating statins and antibiotics, periods in which patients initiated statins were 1:1 matched to non‐use periods based on age (±5 years), gender, calendar date (±60 days), and duration of diabetes in years. Matching was used to be able to determine a start of follow‐up (index date) for non‐use periods among comparable patients. Patients were considered statin initiators if they received a first statin prescription after a period of at least a year without any statin prescription. Non‐use periods were defined as periods where a patient did not receive a statin prescription in the 12 months before or 13 months after the index date. We compared statin initiation periods with non‐use period instead of users versus non‐users to gain more statistical power.^37^, ^38^ Consequently, some patients contribute to both use and non‐use periods, which reduces confounding compared with evaluations where never‐users are used as controls. Follow‐up was started at the date of statin initiation (index date for controls) and stopped at 13 months, similar to the PSSA. When the survival analysis follow‐up period matches the symmetry window and there is no loss to follow‐up, the hazard ratio and sequence ratio should approximate each other.^39^

Stratified (to take into account matching) Cox proportional hazard regression was used to evaluate the risk of receiving antibiotic treatment during periods that statins are used compared with non‐use periods. The following variables were considered as potential (proxies for) risk factors for receiving antibiotic treatment: cardiovascular drugs,^40^ antithrombotic agents, systemic corticosteroids, immunosuppressants, antivirals, antineoplastics, and drugs for obstructive airway diseases, acid‐suppresive drugs, number of antibiotic prescriptions in the previous year, and duration of diabetes. All potential risk factors were included in the final multivariate model. We tested for effect modification by age by assessing the significance of an interaction between statin use and age. Robust variance estimators were used to estimate conservative 95%CI, to account for the fact that some patients contributed more than one episode.^38^ We graphically assessed whether the proportional hazard assumption was met. Statistical analyses were performed using R version 3.0.2. We reported our study in line with a previous paper that pointed out eight important items to enable adequate assessment of the likelihood that a study is affected by unmeasured or residual confounding.^41^

### Sensitivity analyses

It has been hypothesized that although statins seem to have no beneficial influence on the risk of infections in the general population,^5^ statins might lower the risk in certain subgroups.^7^ Therefore, in secondary analysis, we evaluated whether the sequence ratio among patients not treated with any blood‐glucose lowering drugs (ATC code A10)—including patients without diabetes and potentially some diabetic patients that are not treated with glucose‐lowering drugs—prior statin initiation would be more close to 1, indicating no effect.

We also performed two sensitivity analyses using the PSSA. In the first sensitivity analysis, we evaluated whether associations were different for different groups of antibiotics among patients with drug‐treated type 2 diabetes. We estimated the adjusted sequence ratios for the following groups of antibiotics: tetracycline, beta lactam penicillins, sulphonamides and trimethoprim, macrolides, quinolone, and nitrofurantoin.

The PSSA is vulnerable to factors that are both associated with antibiotic use and the timing since statin use. Therefore, we estimated the adjusted sequence ratio using a maximum time‐span of 30 days between first statin and antibiotic prescriptions, thereby limiting the influence of factors that may change over time.

## Results

In total, 4684 patients with drug‐treated type 2 diabetes initiated antibiotics within 13 months of statin initiation. Patients were on average 63 years old (SD 9), 53% was female, 69% had recorded use of cardiovascular drugs, 23% recorded use of antithrombotic agent, 6% recorded use of systemic corticosteroids, and 15% recorded use of drugs for obstructive airway diseases. Immunosuppressants, antivirals, and antineoplastics were used by less than 1% of the patients.

Of all incident statin users, 2513 patients received their first antibiotic prescription before starting statin therapy and 2132 patients started their antibiotic course after initiating statin therapy. These numbers correspond to a crude SR of 0.85 (95%CI 0.80–0.90). After adjusting for trends in prescribing an adjusted sequence ratio (aSR) of 0.86 (95%CI 0.81–0.91) was obtained (Table 1).

**Table 1:** Time‐trend adjusted sequence ratios for the association between statin initiation and antibiotic treatment initiation

Antibiotic (ATC code)	Number of patients initiating antibiotic treatment within 13 months of statin initiation	Crude sequence ratio (95%CI)	Adjusted sequence ratio (95%CI)
Any (J01)	4684	0.84 (0.79–0.89)	0.86 (0.81–0.91)
Tetracycline (J01A)	1316	0.82 (0.73–0.91)	0.86 (0.77–0.96)
Beta lactam penicillin (J01C)	1734	0.87 (0.79–0.96)	0.87 (0.79–0.96)
Sulphonamides and trimethoprim (J01E)	448	0.79 (0.66–0.96)	0.91 (0.76–1.10)
Macrolides (J01F)	360	0.69 (0.56–0.85)	0.70 (0.56–0.86)
Quinolones (J01M)	327	0.95 (0.76–1.18)	0.90 (0.72–1.12)
Nitrofurantion (J01XE)	446	0.96 (0.80–1.16)	0.85 (0.71–1.03)

When the maximum time‐span between both prescriptions was reduced to 30 days the aSR slightly decreased (aSR 0.78, 95%CI 0.64–0.94). Excluding that period from the main analysis resulted in an almost identical sequence ratio (aSR 0.86, 95%CI 0.81–0.92).

The effect was fairly consistent across different types of antibiotics (Table 1). Nitrofurantoin is an antibiotic almost solely used to treat urinary tract infections and may therefore serve as a valid proxy for urinary tract infections.^32^, ^34^ The point estimate for nitrofurantoin was similar as the estimate for all antibiotics (aSR 0.85 vs 0.86).

In secondary analysis, the association between statin initiation and receiving antibiotic prescriptions was much weaker among patients not treated with blood‐glucose lowering drugs (aSR 0.96, 95%CI 0.93 to 0.99).

For the survival analysis, 9852 patients with drug‐treated type 2 diabetes that initiated statin therapy and met inclusion criteria were identified. Subsequently, 4928 patients with drug‐treated type 2 diabetes with non‐use episodes could be matched to the exposed periods. During episodes of statin initiation patients were slightly younger and had a shorter duration of diabetes (Table 2). During follow‐up 3000 first antibiotic prescriptions were observed among the participants. Periods of statin use were associated with a reduced hazard of antibiotic prescriptions compared with non‐use periods (crude HR 0.85, 95%CI 0.79–0.92). Adjusting for all potential confounders resulted in a similar hazard ratio (adjusted HR [aHR] 0.88, 95%CI 0.83–0.95). Other cardiovascular medication and antithrombotic agents were both not associated with a reduction in antibiotic prescriptions in the multivariate cox model (aHR 1.03, 95% 0.92–1.15 and aHR 1.03, 95%CI 0.90–1.17, respectively). The effect of statin initiation was not significantly modified by age (*p* = 0.53). When the patient population was restricted to patients classified as drug‐treated diabetes patients at least one year prior statin initiation, similar results were obtained for statin initiation (aHR 0.91, 95%CI 0.85–0.99). Graphical assessment of the proportional hazard assumption showed that this assumption was met for all covariates.

**Table 2:** Baseline characteristics of patients with drug‐treated type 2 diabetes included in the cohort analysis

	Statin episodes (*n* = 9852)	Non‐use episodes (*n* = 4928)
Mean age in years (SD)	62.3 (9.4)	64.3 (10.1)
Male gender (%)	4922 (50)	2430 (49)
Mean duration of glucose‐lowering drug use in years (SD)	1.5 (2.2)	2.0 (2.2)
Mean number of antibiotics in year prior index date (SD)	0.8 (2.5)	1.0 (3.2)
*Co‐medication (%)*		
Cardiovascular drugs	5906 (60)	3069 (62)
Antithrombotic agents	1873 (19)	1175 (24)
Systemic corticosteroids	537 (6)	382 (8)
Immunosuppressants	104 (1)	72 (2)
Antivirals	27 (0.3)	11 (0.2)
Antineoplastics	24 (0.2)	41 (0.8)
Drugs for obstructive airway diseases	1231 (13)	713 (15)
Acid‐suppressive drugs	2949 (30)	1644 (33)

## Discussion

Statin initiation was associated with a decreased risk of receiving antibiotic prescriptions among patients with drug‐treated type 2 diabetes. Both the PSSA, the unadjusted and adjusted survival analyses gave almost identical results. The similarity between the different designs suggests that the obtained results are not likely because of confounding.

It may seem surprising that the results of the adjusted and unadjusted survival analyses were almost identical. However, non‐use and use periods were very similar with regard to measured risk factors for infections, which can be partly explained by the fact that patients could contribute to both the use and non‐use periods. Moreover, in a previous study that evaluated the effect of statins on the occurrence of pneumonia in patients with diabetes, the crude effect estimate did hardly change after adjustment for a large set of potential confounders.^25^ In that study, patients were matched by practice, which may actually amplify the bias induced by unmeasured confounders, so‐called amplification bias.^42^ Moreover, in that study, prevalent users were compared with never users, which may further bias the results.^43^, ^44^

Hence, our study is relevant, as it is the first study that evaluated whether a protective effect against infections among patients with type 2 diabetes is likely because of time‐invariant unmeasured confounding.

To date, there are no randomized controlled trials available evaluating the effect of statins on the occurrence of infections among patients with drug‐treated type 2 diabetes. As clinical guidelines recommend statin treatment in almost all patients with type 2 diabetes,^45^, ^46^ randomized placebo‐controlled trials evaluating the effect of statins on infections among patients with type 2 diabetes are no longer ethical. Therefore, in the absence of evidence from randomized trials, evidence should be obtained from observational studies that adequately address potential (un)measured confounding. The PSSA we applied does limit unmeasured confounding as we have shown in earlier studies from our group.^32^, ^33^, ^34^

## Strength and Limitations

An important strength of our study is that we evaluated the association of interest using two designs that address confounding in a different way. Despite the differences between both designs, we obtained very similar results, strengthening the confidence in the results. The data were obtained from a widely researched prescription database with proven accuracy of prescription rates among adults.^30^ Furthermore, both statins and antibiotics are not available over the counter in the Netherlands, reducing the likelihood of misclassification.

Our study also has some limitations. First of all, observational studies are well known to be vulnerable to confounding and evidence from such studies is often regarded as less important than evidence from randomized controlled trials.^47^, ^48^ The very similar results obtained with both the PSSA, the unadjusted and adjusted survival analyses suggest that the results are not largely affected by confounding. However, although the PSSA minimizes confounding by factors that are stable over time, we cannot exclude confounding by factors that are both associated with antibiotic use and the timing since statin use. Besides disease severity, which would in fact more likely bias the SR towards an increased risk, we are not aware of any important time‐varying confounders. When the maximum time‐span between both prescriptions was lowered to 30 days, the aSR became slightly lower (0.78 vs 0.86), indicating that if time‐varying confounding would play a role, it would indeed result in an underestimation of the effect.

A second possible limitation is exposure or outcome misclassification. Statin use may be misclassified as some patients may not actually take their drug, despite collecting the drugs from the pharmacy. This could have resulted in an underestimation of the protective effects of statins. Prophylactic use of antibiotics may have had a small impact on our estimates, although a substantial difference in prophylactic use just prior and after statin initiation is unlikely. Some antibiotic use may have been missed, as the database does not capture medication dispensed during hospitalizations.^30^ However, a previous study that evaluated the effect of statins on infections and that did have information about infections treated in hospitals found even larger protective effects,^25^ suggesting that antibiotic prescribing within hospitals could not explain the protective effect. Moreover, the vast majority of antibiotics are prescribed in primary care,^49^ thereby limiting the potential influence of antibiotics prescribed in hospitals.

Finally, the generalizability of our findings of a reduced risk among patients with drug‐treated type 2 diabetes may be restricted to patients with diabetes. The association between statin initiation and receiving antibiotic prescriptions was much weaker among patients not treated with blood‐glucose lowering drugs (aSR 0.96, 95%CI 0.93 to 0.99), consistent with the meta‐analysis of randomized trials that found no effect on the risk of infection (RR 1.00, 95%CI 0.96–1.05).^5^ Unfortunately, that meta‐analysis did not include sufficient patients with diabetes to perform subgroup analysis.

In sensitivity analysis, we observed fairly consistent associations across different type of antibiotics, which suggest that the protective effect of statins may be applicable to various types of infections. However, these results should be interpreted with caution given the reduced numbers per antibiotic group and the fact that the same antibiotic can be used to treat different infections. Nevertheless, the point estimate for nitrofurantoin, as a proxy for urinary tract infections, was similar to a recent study investigating the influence of pravastatin on incident urinary tract infection among adults with persistent microalbuminuria, a condition associated with elevated Rac1 activation,^50^ in a randomized setting (aSR 0.85 vs HR 0.83).^51^ The effect against specific types of infections and the generalizability of our findings to other high‐risk patient groups deserves further investigation.

Given the increasing concern that antimicrobial resistance is a major threat to health care,^52^ additive or alternative treatments are urgently needed. Our data, together with experimental evidence, suggest that statins may reduce the need for antibiotic treatment among patients with diabetes. These findings are relevant, as antibiotic pressure is an important determinant of emergence and dissemination of antibiotic resistance.^52^ Moreover, if it can be proven that statins indeed reduce the risk of infections, patients with diabetes may have another good reason to improve their adherence to statin treatment as infections are common among patients with diabetes. Improved statin adherence among patients with diabetes may reduce antibiotic usage, prevent cardiovascular events, and improve the cost‐effectiveness of statin treatment. However, in the absence of randomized controlled trials, further conformation using other confounding minimizing designs and analytical methods is needed.

In conclusion, statin initiation was associated with a reduced risk of receiving antibiotic prescriptions among patients with drug‐treated type 2 diabetes. Our results support the hypothesis that statins might be helpful in preventing infections and reduce antibiotic usage among this group of patients.

## Funding

This study was carried out as part of our routine work.

## Prior Postings and Presentations

The manuscript has not been published and is not being considered for publication elsewhere, in whole or in part, in any language. An abstract of this manuscript has been presented orally at the 4th International Conference on Pharmacy and Advanced Pharmaceutical Sciences (ICPAPS 2015), Indonesia.

## Conflict of Interest

The authors declare no conflict of interest

**Key Points**

- The present findings suggest that statins are associated with a reduced risk of infections among patients with drug‐treated type 2 diabetes.
- Almost identical results were obtained using the cohort and prescription sequence symmetry analysis, suggesting that results are not largely influenced by time‐invariant counfounding

## Ethics Statement

Ethical approval was not required to perform this study.
